# False-positive reduction in mammography using multiscale spatial Weber law descriptor and support vector machines

**DOI:** 10.1007/s00521-013-1450-7

**Published:** 2013-07-13

**Authors:** Muhammad Hussain

**Affiliations:** Department of Software Engineering, College of Computer and Information Sciences, King Saud University, Riyadh, Saudi Arabia

**Keywords:** WLD, Support vector machines, Mass detection, Mammograms, False-positive reduction

## Abstract

In a CAD system for the detection of masses, segmentation of mammograms yields regions of interest (ROIs), which are not only true masses but also suspicious normal tissues that result in false positives. We introduce a new method for false-positive reduction in this paper. The key idea of our approach is to exploit the textural properties of mammograms and for texture description, to use Weber law descriptor (WLD), which outperforms state-of-the-art best texture descriptors. The basic WLD is a holistic descriptor by its construction because it integrates the local information content into a single histogram, which does not take into account the spatial locality of micropatterns. We extend it into a multiscale spatial WLD (MSWLD) that better characterizes the texture micro structures of masses by incorporating the spatial locality and scale of microstructures. The dimension of the feature space generated by MSWLD becomes high; it is reduced by selecting features based on their significance. Finally, support vector machines are employed to classify ROIs as true masses or normal parenchyma. The proposed approach is evaluated using 1024 ROIs taken from digital database for screening mammography and an accuracy of Az = 0.99 ± 0.003 (area under receiver operating characteristic curve) is obtained. A comparison reveals that the proposed method has significant improvement over the state-of-the-art best methods for false-positive reduction problem.

## Introduction

Breast cancer is one of the most common types of cancer among women all over the world, and it is considered as the second main cause of death among women [1]. According to a survey conducted by the American Cancer Society, one out of 8–12 American women will suffer from breast cancer during his lifetime [2]. Also, 19 % European women out of those suffering from breast cancer die due to this type of cancer [3]. Moreover, the World Health Organization’s International Agency for Research on Cancer (IARC) reported that 0.4 million women die every year due to breast cancer out of more than one million registered cases of breast cancer [4]. The detection of breast cancer at an early stage can be effective in preventing deaths due to breast cancer, but it is not an easy task. Commonly used imaging modality for breast cancer is mammogram, which has significantly enhanced the radiologists’ ability to detect and diagnose cancer at an early stage and take immediate precautions for its earliest prevention [5].

The analysis of mammograms is a complicated task due to its complex structure. The malignant abnormalities found through mammogram screening are about 0.1–0.3 % [6]. In addition, after follow-up mammograms, only 5–10 % of the suspected abnormalities are recommended for surgical verification by biopsy [7] and about 60–80 % biopsies result in false positives [8]. On the other hand, retrospective analysis reveals false-negative rate of 10–20 % [8]. It follows that a significant number of abnormalities is missed by expert radiologists. Given the number of mammograms screened every year, a small decrease in false negatives can save many lives and a small decrease in false positives can result in significant reduction in unnecessary follow-ups and mental trauma.

Mammography provided an opportunity to introduce computer-aided detection (CAD) systems in order to help the radiologists for detecting and diagnosing the breast cancer at an early stage [9–11]. In 2001, Freer and Ulissey [12] evaluated a CAD system for 12,860 patients and concluded that CAD system can improve the detection of malignant cases in their early stages. However, this fact became controversial in 2005 when Khoo et al. [13] published their results for a database of 6111 women. Nishikawa and Kallergi [12] argued that CAD in its present form does not have significant impact on the detection of breast cancer. The main reason for the mistrust of radiologists on the role of CAD system in breast cancer detection is due to large number of false positives [8, 14].

In a CAD system for masses, mammograms are segmented to detect masses; the segmentation yields regions of interest (ROIs), which are not only masses but suspicious normal tissues as well, which result in false positives. The performance of a CAD system depends on how much accurately the false positives are reduced. The reduction in false positives is dependent on the description of ROIs. Various descriptors based on texture, gray level, ICA [15, 21], PCA [16], 2DPCA [17, 18], morphology [19], wavelets [20], and LBP [21] have been used. Lladó et al. [21] used spatially enhanced local binary pattern (LBP) descriptor, which is basically a texture descriptor, to represent textural properties of masses and to reduce false positives; this method achieved an overall accuracy of Az = 0.94 ± 0.02 (percentage area under receiver operating characteristic (ROC) curve) on digital database for screening mammography (DDSM). This is the best of all false-positive reduction methods published so far. But LBP descriptor builds statistics on local micropatterns (dark/bright spots, edges, and flat areas) without taking into account the directional information of texture micropatterns; also, it is not robust against noise. Instead of LBP, we use Weber law descriptor (WLD) [22] for representing the textural properties of masses and to reduce the false positives. WLD builds statistics on salient micropatterns along with gradient orientation of the current pixel and is robust against noise and illumination changes. Chen et al. [22] have shown that WLD outperforms LBP in texture recognition. As such, WLD is a better choice for representing the texture properties of masses and normal parenchyma.

The basic WLD is a histogram where differential excitation values are integrated according to their gradient orientations irrespective of their spatial location and so WLD behaves like a holistic descriptor. We extend it to enhance its discriminatory power by embedding the spatial locality and the scale of micropatterns that better characterize the spatial structures of masses; we call it multiscale spatial WLD (MSWLD), initially employed in [30]. The main contributions of the paper are as follows:

(i) Effective representation of mass and normal ROIs using multiscale spatial WLD (MSWLD).
(ii) Finding the best set of the values of the parameters of MSWLD that results in the best representation of masses and normal ROIs.
(iii) Selection of the significant features in MSWLD.
(iv) A false-positive reduction method for a CAD system of masses based on MSWLD and support vector machine (SVM) that significantly reduces false positives.

The organization of the rest of the paper is as follows. Section 2 illustrates the main algorithms for false-positive reduction problem. Section 3 presents the architecture of the system for false-positive reduction and the description of the database used for the validation of the system. Results have been reported and discussed in Sect. 4. Section 5 concludes the paper.

## Materials and methods

In this section, first we give an overview of the basic WLD [22] and its multiscale version. Then, we describe its extensions—spatial WLD (SWLD) and multiscale spatial WLD (MSWLD). This descriptor represents an image as a histogram of differential excitations, according to the corresponding gradient orientations, and has several interesting properties like robustness to noise and illumination changes, elegant detection of edges, and powerful image representation. These characteristics have made it suitable for detection tasks involving complex texture patterns with varying conditions.

Weber law descriptor is based on Weber’s Law. According to this law, the ratio of the increment threshold to the background intensity is constant. Inspired by this law, Chen et al. [22] proposed WLD for texture representation. The computation of WLD involves three components: calculating differential excitations, gradient orientations, and building the histogram. In the following sections, first we give an overview of these components and then the detail of MSWLD is presented.

### Differential excitation (DE)

**Fig. 1:**
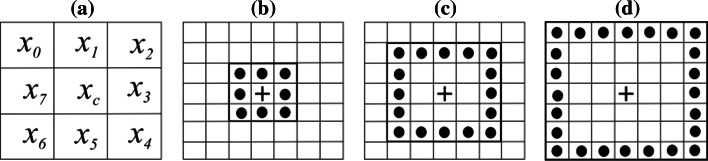
**a** Central pixel and its neighbors in case *P* = 8. **b** (8, 1) neighborhood of the central pixel, **c** and **d** (16, 2) and (24, 3) neighborhoods, respectively, of the central pixel [22]

The first step for WLD is the computation of the differential excitation (DE) of each pixel. To compute DE ε(*x*_*c*_) of a pixel *x*_*c*_, first intensity differences of *x*_*c*_ with its neighbors *x*_*i*_, *i* = 0, 1, 2, …, *p* − *1* (see Fig. 1a for the case *p* = 8) are calculated as follows [22]:

1 $$\Delta I_{i} = I_{i} - I_{c} .$$

Then, the ratio of the total intensity difference $\sum\nolimits_{i = 0}^{P - 1} {\Delta I_{i} }$ to the intensity of *x*_*c*_ is determined as follows:

2 $$f_{\text{ratio}} = \mathop \sum \limits_{i = 0}^{P - 1} \left( {\frac{{\Delta I_{i} }}{{I_{c} }}} \right).$$

Note that *f*_ratio_ is not robust against noise. Arctangent function is applied on *f*_ratio_ to enhance the robustness of WLD against noise, which finally gives the DE for pixel *x*_*c*_:

3 $$\varepsilon \left( {x_{c} } \right) = { \arctan }\left[ {\mathop \sum \limits_{i = 0}^{P - 1} \left( {\frac{{\Delta I_{i} }}{{I_{c} }}} \right)  } \right].$$

The differential excitation ε(*x*_*c*_) may be positive or negative. If the current pixel is darker than its background, then its gray scale value *I*_*c*_ is less than those (*I*_*i*_, *i* = 0, 1, 2, …, *P*−1) of its neighbors and each $\Delta I_{i}$ is positive. As such, the positive value of DE means that the current pixel is darker than its background and the negative value of DE indicates that the current pixel is lighter than its background.

### Gradient orientation (GO)

Next main component of WLD is gradient orientation. For a pixel *x*_*c*_, the gradient orientation is calculated as follows [22]:

4 $$\theta \left( {x_{c} } \right) \, = \arctan \left[ {\frac{{I_{73} }}{{I_{51} }}} \right]$$

where *I*_73_ = *I*_7_ – *I*_3_ is the intensity difference of two pixels on the left and right of the current pixel *x*_*c*_, and $I_{51} = I_{5} - I_{1}$ is the intensity difference of two pixels directly below and above the current pixel, see Fig. 1a. Note that $\theta \in \left[ { - \frac{\pi }{2}, \frac{\pi }{2}} \right]$.

The gradient orientations are quantized into *T* dominant orientations as:

5 $$\phi_{t} = \frac{2t}{T}\pi  \quad  {\text{where}} \;t = \bmod \left( {\left\lfloor {\frac{{\theta^{\prime}}}{2\pi /T} + \frac{1}{2}} \right\rfloor ,T} \right)$$

where $\theta^{\prime} \in [0, 2\pi ]$ and is obtained using the mapping *f*: *θ* → *θ′* defined in terms of gradient orientation computed by the Eq. (4) as follows:

$$\theta^{\prime} = { \arctan }\,2\left( {I_{73} ,  I_{51} } \right) + \pi$$

where

$$\arctan \,2\left( {I_{{73}} ,I_{{51}} } \right) = \left\{ {\begin{array}{*{20}c}    \theta  \hfill & {I_{{73}}  > 0\,\,{\text{and }}\:I_{{51}}  > 0} \hfill  \\    {\pi  + \theta } \hfill & {I_{{73}}  > 0\,\,{\text{and }}\:I_{{51}}  > 0} \hfill  \\    {\theta  - \pi } \hfill & {I_{{73}}  < 0\,\,{\text{and}}\;I_{{51}}  < 0} \hfill  \\    \theta  \hfill & {I_{{73}}  < 0\,\,{\text{and}}\;I_{{51}}  < 0} \hfill  \\   \end{array} } \right.$$

In case *T* = 8, the dominant orientations are $\phi_{t} = \frac{t\pi }{4} , t = 0,1, \ldots ,T - 1$; all orientations located in the interval $\left[ { \phi_{t} - \left( {\frac{\pi }{8}} \right), \phi_{t} + \left( {\frac{\pi }{8}} \right)} \right]$ are quantized as *ϕ*_*t*_.

### Basic WLD

**Fig. 2:**
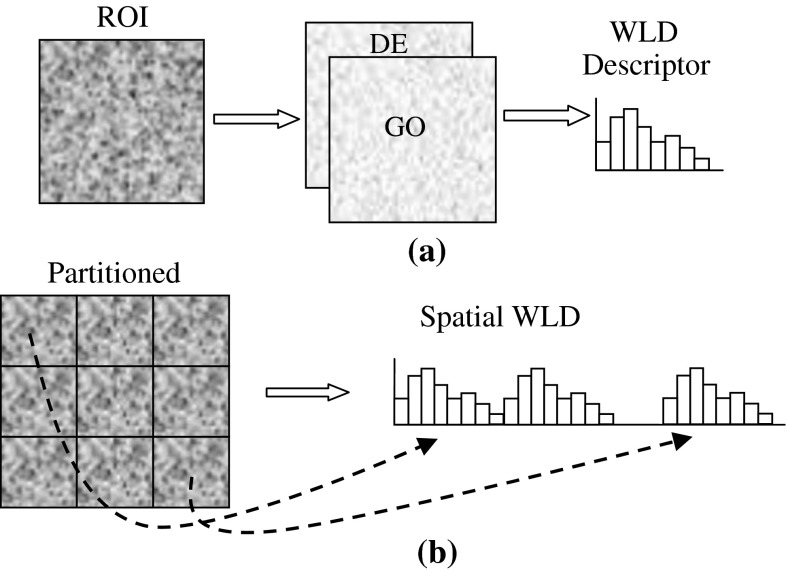
**a** Basic WLDr, **b** spatial WLD

The differential excitation and dominant orientation calculated for each pixel form a WLD feature. Using these features, WLD histogram is calculated, see Fig. 2a. First, sub-histograms *H*_*t*_: *t* = 0, 1, 2, …, *T−*1 of differential excitations corresponding to each dominant orientation *ϕ*_*t*_: *t* = 0, 1, 2, …, *T*−1 are calculated; all pixels having dominant direction, *ϕ*_*t*_, contribute to sub-histogram *H*_*t*_. Then, each sub-histogram *H*_*t*_: *t* = 0, 1, 2, …, *T*−1 is further divided into *M* sub-histograms *H*_*m,t*_: *m* = 0, 1, 2, …, *M*−1, each with S bins. These sub-histograms form a histogram matrix *H*_*m,t*_: *m* = 0, 1, 2, …, *M*−1, *t* = 0, 1, 2, …, *T*−1, where each column corresponds to a dominant direction *ϕ*_*t*_. Each row of this matrix is concatenated as a sub-histogram *H*_*m*_ = {*H*_*m,t*_: *t* = 0, 1, 2, …, *T*−1}. Subsequently, sub-histograms *H*_*m*_: *m* = 0, 1, 2, …, *M*−1 are concatenated into a histogram *H* = {*H*_*m*_: *m* = 0, 1, 2, …, *M*−1}. This histogram represents an image and is referred to as WLD. This descriptor involves three free parameters:

- *T*, the number of dominant orientations *ϕ*_*t*_: *t* = 0, 1, 2, …, *T*−1,
- *M,* the number of segments *H*_*m,t*_ of each sub-histogram *H*_*t*_ corresponding to a dominant orientation *ϕ*_*t*_, and
- *S*, the number of bins in each sub-histogram *H*_*m,t*_.

We represent basic WLD operator by WLD (*T*, *M*, *S*).

### Multiscale WLD

The WLD reviewed in the previous sections uses fixed size 3 × 3 neighborhood, see Fig. 1a and is unable in characterizing local salient patterns in different granularities. For representing local salient patterns at different scales, it is extended to multiscale WLD, which is computed using a symmetric square neighborhood (*P, R*) of side (2*R* + 1) centered at the current pixel and consisting of *P* pixels along the sides of the square. The neighborhoods (*P, R*)—*R* = 1, 2, 3 and *P* = 8, 16, 24—determine the scale of the descriptor [22]. For multiscale analysis, histograms obtained using WLD operators with varying (*P*, *R*) neighborhoods are concatenated. We represent multiscale WLD operator by MWLD_*P,R*_ (*T, M, S*).

### Spatial WLD

WLD feature is a local feature but WLD histogram is a holistic descriptor that represents an image as a histogram of differential excitations. In this histogram, differential excitations are put into bins according to their values and gradient orientations, irrespective of their spatial location. In this way, locally salient patterns might be lost when an image, such as a mammogram, has different texture patterns at different locations. Spatial location is also an important factor for better description. For example, two similar structures occurring in two different patterns having different spatial locations will contribute to the same bins in the histogram and will not be discriminated by WLD. To enhance the discriminatory power of WLD, we incorporate spatial information into the descriptor. Each image is divided into a number of blocks *B*_*1*_ *, B*_*2*_, …, *B*_*n*_, WLD histogram *H*_*Bi*_ is computed for each block and then these histograms are concatenated to form a Spatial WLD (SWLD) *H* = {*H*_*Bi*_: *i* = 1, 2, …, *n*}. SWLD not only encode gradient orientation information but also the spatial locality of salient micropatterns.

This descriptor has better discriminatory power because it captures the spatial locality of micropatterns in a better way, which is important for recognition purpose. This extension introduces another parameter: the number of blocks. The suitable choice of number of blocks can lead to better recognition results. We specify SWLD operator by SWLD (*T*, *M*, *S, n*), where *n* is the number of blocks.

### Multiscale spatial WLD

Spatial WLD characterizes both directional and spatial information at fixed granularity. For better representation of an image, it is important to capture local micropatterns at varying scales (*P*, *R*). To achieve this end, we introduce MSWLD; in this case for each block of an image, a multiscale WLD histogram at a particular scale (*P*, *R*) is computed and then these histograms are concatenated. The final histogram is the MSWLD at scale (*P*, *R*). We represent multiscale spatial WLD operator by MSWLD_*P,R*_ (*T*, *M*, *S, n*).

Note that the multiscale WLD proposed in [22] is realized with MWLD_*P,R*_ (*T*, *M*, *S*) operator, whereas the proposed MSWLD is computed using MSWLD_*P,R*_ (*T*, *M*, *S, n*) operator.

### Significance of features

The dimension of the feature space generated by MSWLD becomes excessively high. All features are not significant. The redundant features not only increase the dimension of the feature space—curse of dimensionality—but also create confusion for the classifier and result in the decrease in classification accuracy. There is the need to select the most significant features. Different methods can be used to identify irrelevant features and select only the most significant ones. We employ the method proposed by Sun et al. [23]. This method is simple, powerful, and robust; its detail is given below.

Let *D* = {(*x*_*i*_, *y*_*i*_) : *i* = 1, 2, …, *n*} be a training dataset, where *x*_*i*_ ϵ *R* ^*m*^ and *y*_*i*_ ϵ {±1} are the feature descriptor and class label of *i*th training sample. Let *w* be an *m*-dimensional nonnegative weight vector whose components represent the relevance of the corresponding *m* features of *x*_*i*_. The problem of feature subset selection is to compute *w* so that a margin-based error function in the weighted feature space parameterized by *w* is minimized, which is an arbitrary nonlinear problem. This problem is solved iteratively in two stages. First, by local learning, this problem is decomposed into locally linear problems of learning margins (Steps 3 and 4 in the following pseudocode). Then, *w* is learned within large margin framework based on logistic regression formulation (Step 5 in the following pseudocode).

The pseudocode of the algorithm is given below [23].



In this algorithm,

$$\varvec{z}_{i} = \mathop \sum \limits_{{\varvec{r} \in M_{i} }} P(\varvec{x}_{r} = NM(\varvec{x}_{i} )|w)\left| {\varvec{x}_{i} - \varvec{x}_{r} } \right| - \mathop \sum \limits_{{\varvec{r} \in H_{i} }} P(\varvec{x}_{r} = NH(\varvec{x}_{i} )|w)\left| {\varvec{x}_{i} - \varvec{x}_{r} } \right|$$

where $M_{i} = \{ r:1 \le r \le n, y_{r} \ne y_{i} \}$, $H_{i} = \{ r:1 \le r \le n, y_{r} = y_{i} , r \ne i\}$, $P\left( {\varvec{x}_{r} = NM\left( {\varvec{x}_{i} } \right) |\varvec{w}} \right) = \frac{{{ \exp }\left( {\left\| {\varvec{x}_{i} - \varvec{x}_{r} } \right\|_{w} /\sigma } \right)}}{{\mathop \sum \nolimits_{{s \in M_{i} }} { \exp }\left( {\left\| {\varvec{x}_{i} - \varvec{x}_{s} } \right\|_{w} /\sigma } \right)}}, \forall r \in M_{i}$, $P\left( {\varvec{x}_{r} = NH\left( {\varvec{x}_{i} } \right) |\varvec{w}} \right) = \frac{{{ \exp }\left( {\left\| {\varvec{x}_{i} - \varvec{x}_{r} } \right\|_{w} /\sigma } \right)}}{{\mathop \sum \nolimits_{{s \in H_{i} }} { \exp }\left( {\left\| {\varvec{x}_{i} - \varvec{x}_{s} } \right\|_{w} /\sigma } \right)}}, \forall r \in H_{i}$, *NM* (*x*_*i*_) denotes the nearest neighbor of *x*_*i*_ belonging to the opposite class, *NM*(*x*_*i*_) represents the nearest neighbor of *x*_*i*_ belonging to its class, and the kernel width *σ* is a free parameter that determines the resolution at which the data are locally analyzed. The regularization parameter *λ* controls the sparseness of the solution and *η* is the learning rate. For further detail, a reader is referred to [23].

**Fig. 3:**
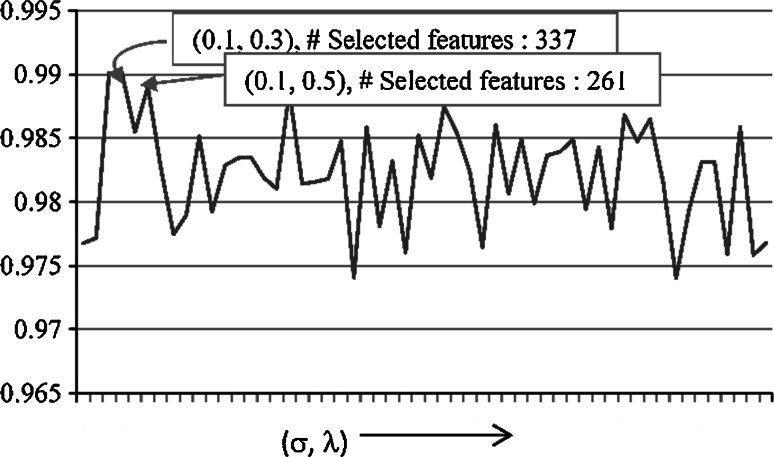
Graph showing the effect of the parameters (*σ*, *λ*) on classification accuracy



This method has two free parameters: kernel width *σ* and regularization parameter *λ*. Though the authors claim in [23] that the performance of the method does not depend on a particular choice of the values of these parameters, our experience is different, see Fig. 3; the proper choice of these parameters is imperative for the best results. To find the optimal values of *σ* and λ, which help to select the minimum number of the most significant features giving the best classification result, we applied grid search, as described below.

Though Sun’s method is a filter method but we employed it as a wrapper method for feature subset selection.

### Support vector machine (SVM)

**Fig. 4:**
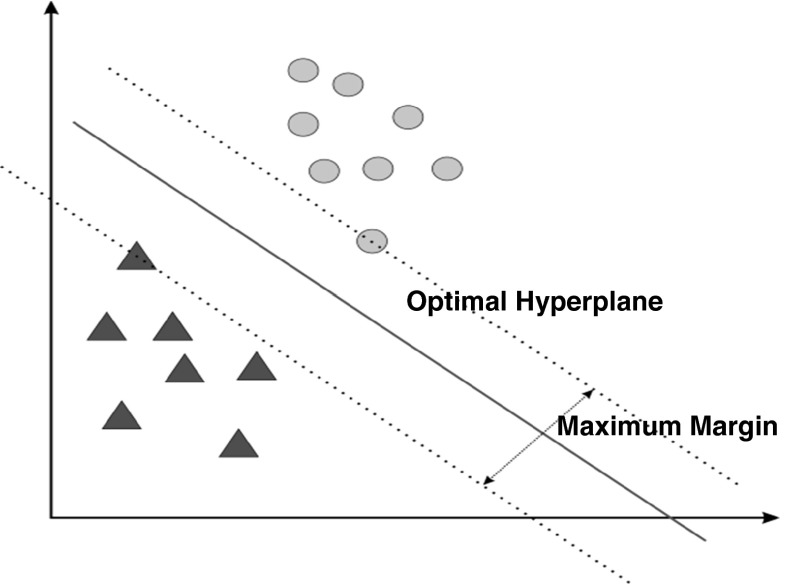
SVM classifies by finding the optimal hyperplane that has maximum margin

For classification, support vector machines (SVM) [24] are used; it is one of the most advanced classifier and outperforms other well-known classification methods in many applications involving two-class problem, especially in texture classification problem. The interesting aspect of SVM is its better generalization ability that is achieved by finding optimal hyperplane with maximum margin, see Fig. 4. The optimal hyperplane is learned from training set. More specifically, given the training samples {(*x*_*i*_, *y*_*i*_): *i* = 1, 2, …, *n*}, where *x*_*i*_ and *y*_*i*_ ϵ {−1, +1} are the feature descriptor and class label of *i*th training sample, the optimal hyperplane is defined as follows:

$$f(x) = \varvec{w}.\varvec{x} + b = 0$$

where ***w*** and *b* are obtained by solving the following optimization problem:

$$\begin{array}{*{20}c} {\text{Minimize}} & {\frac{1}{2}\left\| \varvec{w} \right\|^{2} } \\ {\text{Subject to the constraints}} & {y_{i} \left( {\varvec{w}.\varvec{x}_{i} + b} \right) \ge 1, i = 1, 2, \ldots , n} \\ \end{array}.$$

The solution of this problem ensures that the margin $\frac{2}{{\left\| \varvec{w} \right\|}}$ of the hyperplane is maximum. The training samples that are on the canonical hyperplanes (***w***.***x*** + *b*) = ±1 are known as support vectors. Note that *y*_*i*_ = 1 for a normal ROI and *y*_*i*_ = −1 for a mass ROI.

Support vector machines are basically a linear classifier that classifies linearly separable data, but in general, the feature vectors might not be linearly separable. To overcome this issue, kernel trick is used. Using a kernel function that satisfies Mercer’s condition, the original input space is mapped into a high-dimensional feature space where it becomes linearly separable. Using kernel trick, the general form of an SVM is

$$f(\varvec{x}) = \mathop \sum \limits_{{\varvec{i} \in \Upomega }} \alpha_{i} y_{i} K(\varvec{x},\varvec{x}_{\varvec{i}} ) + b$$

where $\alpha_{i}^{'} s$ are Lagrange coefficients due to Lagrange formulation of the optimization problem, **Ω** is the set of indices of nonzero $\alpha_{i}^{'} s$, which corresponds to the support vectors, ***x*** is a testing sample, and *K* (***x***, ***x***_*i*_) is a kernel function. Classification decision is taken based on whether *f*(*x*) as a value above or below a threshold. Different kernel functions have been employed for different classification tasks. As radial basis function (RBF) gives the best results in most of the applications, we employ RBF for false-positive reduction problem. SVM with RBF kernel involves two parameters: C, the penalty parameter of the error term and γ, the kernel parameter. For optimal classification results, these parameters must be properly tuned. We select the optimal values of these parameters using first coarse and then fine grid search. For implementation of SVM, we used LIBSVM [25].

## False-positive reduction system

**Fig. 5:**
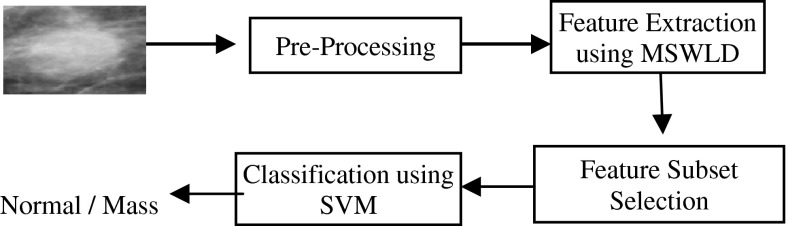
Mass detection system

The block diagram of the false-positive reduction system is shown in Fig. 5. There are four main components of the system: preprocessing, feature extraction, feature selection, and classification. Various existing approaches differ in the choice of techniques for these components. Note that WLD is robust against noise and illumination changes [22], so in our approach there is no need for preprocessing methods for denoising and enhancement. For feature extraction, we used MSWLD, which has been discussed in detail in Sect. 2. The method proposed by Sun et al. [23] is used for selecting the most significant features, and SVM with RBF is employed for classification. The novelty of the system is to use a powerful discriminating MSWLD along with feature selection for reducing the number of false positives.

### Database

**Fig. 6:**
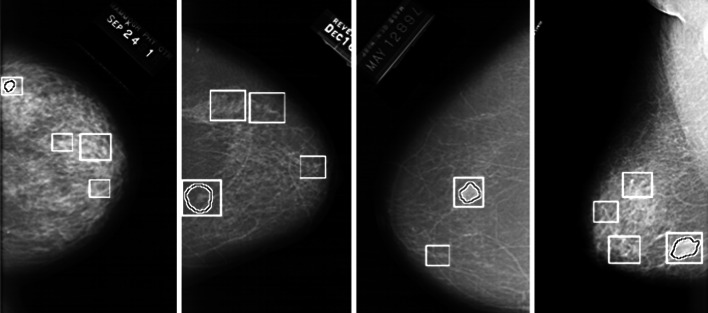
Annotated mammogram images from DDSM database. Contours mark the boundaries of the mass regions. *Squares* represent the mass and suspicious normal ROIs extracted for the validation of the proposed method

**Fig. 7:**
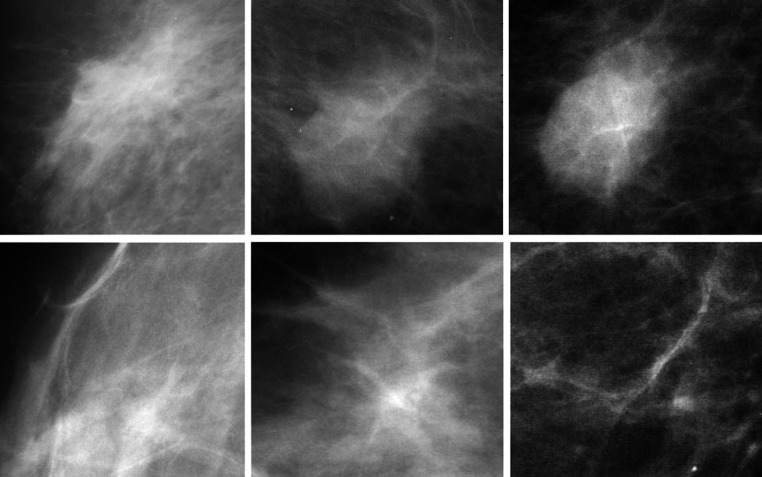
Sample mass ROIs (*top row*) and suspicious normal ROIs (*bottom row*)

The proposed method is evaluated using DDSM [26]; this database consists of more than 2000 cases and is commonly used as a benchmark for testing new proposals dealing with processing and analysis of mammograms for breast cancer detection. The mammograms of the DDSM database were digitized using different scanners: a DBA M2100 ImageClear (42 × 42 μm pixel resolution), a Howtek 960 (43.5 × 43.5 μm pixel resolution), a Lumisys 200 Laser (50 × 50 μm pixel resolution), and a Howtek MultiRad850 (43.5 × 43.5 μm pixel resolution). All the images are 16 bits per pixel. Finally, we rescaled the images to have the same resolution: 50 μm. Each case in this database is annotated by expert radiologists; the complete information is provided as an overlay file. The locations of masses in mammograms specified by experts are encoded as code chains; in Fig. 6, the contours drawn using code chains enclose the true masses. We randomly selected 250 mammograms of the patients, which contain proven true masses, and extracted 1024 ROIs (normal and mass) from these mammograms, see Fig. 6. We extracted 256 ROIs, which contain true masses using code chains; the sizes of these ROIs vary depending on the sizes of the mass regions from 267 × 274 to 1197 × 1301 pixels. In addition, suspicious normal ROIs, which look like masses and result in false positives, were extracted. Some sample ROIs are shown in Fig. 7. These ROIs are uvnsed for training and testing. In an automatic system, it is assumed that these ROIs are extracted by some detection and segmentation algorithm. The role of the proposed algorithm is to identify whether an ROI is a true mass or a normal tissue.

## Experiments and discussion

In this section, we report and discuss the results of the proposed method. For validation, we used DDSM database and directly compared the proposed method with state-of-the-art best similar mass detection method proposed by Llado et al. [21] using the same hardware and software environment and the dataset. In the following subsections, first we describe evaluation strategy, then discuss the impact of the parameters of MSWLD, and finally give the comparison.

### Evaluation strategy

For the evaluation of classification performance, we used fivefold cross-validation. In particular, the dataset is randomly partitioned into five nonoverlapping and mutually exclusive subsets. For the experiment of fold *i*, subset *i* is selected as testing set and the remaining four subsets are used to train the classifier, i.e., 80 % of the dataset is used for training the system and the remaining 20 % samples are used to test the system. The experiments are repeated for each fold and the mean performance is reported. Using fivefold cross-validation, the performance of the method can be confirmed against any kind of bias involved in the selection of the samples for training and testing phases. It also helps in determining the robustness of the method when tested over different ratios of normal and abnormal ROIs used as training and testing sets (due to random selection, ratios will be different). To compute the best parameters (σ, λ) of the Sun’s algorithm, we used fivefold cross-validation and the wrapper approach described in Sect. 2.7.

Commonly used evaluation measures of the predictive ability of a classification method are *sensitivity* (a measure of true-positive rate), *specificity* (a measure of true-negative rate), *accuracy* and *area under ROC curve* (AUC or Az). The sensitivity is defined by

$${\text{Sensitivity}} = \frac{\text{TP}}{{{\text{TP}} + {\text{FN}}}}$$

where TN is the number of ROIs correctly classified as true masses and FN is the number of ROIs, which are wrongly classified as masses. The specificity is defined by

$${\text{Specificity}} = \frac{\text{TN}}{{{\text{TN}} + {\text{FP}}}}$$

where TN is the number of ROIs correctly classified as normal and FP is the number of mass ROIs, which are wrongly classified as normal ROIs. The accuracy is defined by

$${\text{Accuracy}} = \frac{{{\text{TP}} + {\text{TN}}}}{{{\text{TP}} + {\text{TN}} + {\text{FP}} + {\text{FN}}}},$$

it expresses the overall rate of correctly classified ROIs. Another performance measure to evaluate the ability of a classification system to differentiate normal ROIs from mass ROIs is the area (Az) under the ROC curve. The ROC curve describes the ability of the classifiers to correctly differentiate the set of ROIs into two classes based on the true-positive fraction (sensitivity) and false-positive fraction (1 − specificity).

Accuracy is a function of sensitivity and specificity, and it is common trend to use this measure for overall performance of a mass classification method, but a study by Huang and Ling [27] showed that Az is a better measure than *accuracy.* In view of this, our analysis of performance will mainly be based on Az.

### Optimization of parameters

The MSWLD operator—MSWLD_*P,R*_ (*T*, *M*, *S, n*)—involves 6 parameters: *T*, *M*, *S*, the number of blocks *n*, and the scale parameters (*P*, *R*). The recognition rate depends on the proper tuning of these parameters. In this subsection, we discuss the impact of these parameters and describe the optimal combination that yield the best recognition accuracy in terms of Az.

#### Effect of *T*, *M*, and *S*

**Fig. 8:**
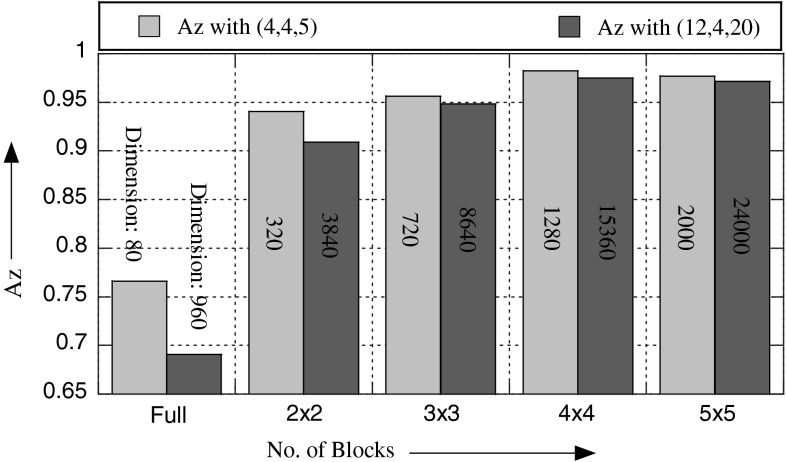
The effect of the combinations (4, 4, 5) and (12, 4, 20), and different number of blocks with MSWLD_24,3_ (*T*, *M*, *S, n*) operator at scale (24, 3). In each case, the dimension of the feature space is shown on bars

To assess the effect of *T*, *M*, *S* on the recognition accuracy, we consider MSWLD operator—MSWLD_24,3_ (*T*, *M*, *S, n*), apply it with different combinations (*T*, *M*, *S*) of *T* = 4, 6, 8, 12; *M* = 4, 8; and *S* = 5, 10, 15, 20 on ROIs with different numbers of blocks and extract MSWLD at scale (24, 3) and use them for mass detection; why we have chosen the scale (24, 3) will be made clear under the discussion of scale parameters. Among different combinations, here we present the results only for two best combinations: (4, 4, 5) and (12, 4, 20); the obtained recognition rates (in terms of Az) in these two cases are shown in Fig. 8 and Table 1. It is obvious that there are no significant differences between Az values obtained for different numbers of blocks. The Az values for the case (4, 4, 5) are bit higher than those for (12, 4, 20). In the first case, the dimension of the feature space is much smaller than that in the second case, look at the bars in Fig. 8. It means that (4, 4, 5) is the best choice. In all our experiments, we will use this combination.

**Table 1:** Effect of combinations of (*T*, *M*, *S*) and block sizes

Number blocks	(*T*, *M*, *S*)	Sensitivity	Specificity	Accuracy	Az
4 × 4	(4, 4, 5)	**98.45** **±** **1.33**	**97.56** **±** **1.23**	**98.00** **±** **0.56**	**0.98** **±** **0.006**
	(12, 4, 20)	98.02 ± 1.59	96.68 ± 0.66	97.36 ± 0.74	0.97 ± 0.009
5 × 5	(4, 4, 5)	98.25 ± 0.85	97.45 ± 0.83	97.85 ± 0.76	0.97 ± 0.008
	(12, 4, 20)	97.88 ± 2.17	96.28 ± 2.24	97.07 ± 1.97	0.97 ± 0.02

Bold values indicate the best results

#### Effect of scales (*P*, *R*)

Three scales are used for experiments: scale-1: (8, 1), scale-2: (16, 2), and scale-3: (27, 3). Figures 6 and 7 show the recognition rates with these three scales and their fusion.

The bar graphs in these figures indicate that scale-3 gives the best recognition performance in terms of Az.

#### Effect of number of blocks and feature selection

**Fig. 9:**
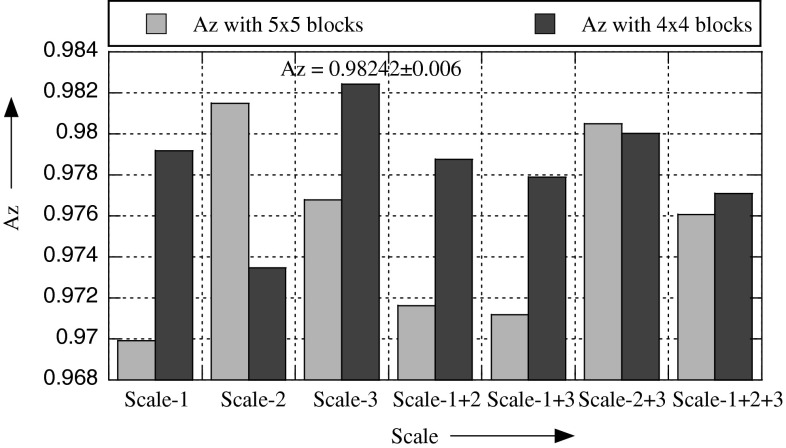
The effect of scale-1: (8, 1), scale-2: (16, 2), scale-3: (27, 3), and their fusion on the recognition rate before feature selection

**Fig. 10:**
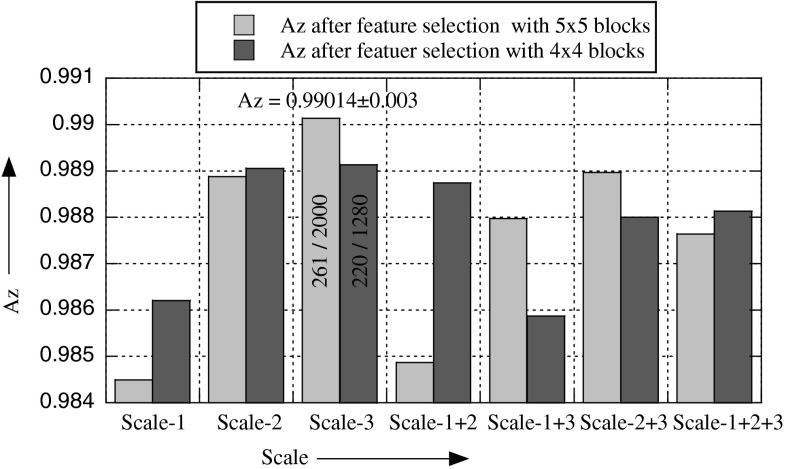
The effect of scale-1: (8, 1), scale-2: (16, 2), scale-3: (24, 3), and their fusion on the recognition rate after feature selection. The numbers on *two bars* show the number of features (*after*/*before*) selection

To find the optimal number of blocks, we performed experiments by dividing each ROI into 1 × 1 (full), 2 × 2, 3 × 3, 4 × 4, and 5 × 5 blocks, i.e., 1, 4, 9, 16, and 25 blocks. From Fig. 9, it is clear that 4 × 4 and 5 × 5 give similar recognition rates, but in case of 5 × 5, the dimension of the feature space becomes very big. It means that the best choice is 4 × 4. It is also obvious from Fig. 9 and Table 2, the recognition rate is maximum (Az = 0.9827 ± 0.006) when 16 (4 × 4) blocks are used. This is the conclusion before feature selection. But after feature selection, the situation is different; the best result (Az = **0.9901** **±** **0.003**) is obtained when 25 (5 × 5) blocks are used, see Fig. 10 and Table 2. In case of 4 × 4 blocks, the number of features before and after selection is 1280/220, whereas this number is 2000/261 when 5 × 5 blocks are used. Also compare the recognition rate before and after feature selection; it is Az = 0.9827 ± 0.006/Az = 0.9891 ± 0.002, and Az = 0.97678 ± 0.008/Az = 0.9901 ± 0.003 before/after feature selection in case of 4 × 4 and 5 × 5 blocks, respectively. It indicates that there is a large number of irrelevant features in the descriptor, which cause confusion for the classifier; when these features are removed by the feature selection algorithm by selecting significant features, the recognition rate has improved significantly. It follows from the above discussion that the best results are obtained with MSWLD_24,3_ (4, 4, 5, 5 × 5) and MSWLD_24,3_ (4, 4, 5, 4 × 4) operators.

**Table 2:** Performance with 4 × 4 and 5 × 5 blocks before and after feature selection

Number blocks	Number features	Sensitivity	Specificity	Accuracy	Az	(*C*, *γ*)	(*σ*, *λ*)
4 × 4	1280	98.45 ± 1.33	97.56 ± 1.23	98.00 ± 0.56	0.98 ± 0.006	(2^9^, 2^−17^)	
	220 (A. F. S.)	99.02 ± 0.47	98.14 ± 0.39	98.58 ± 0.26	0.98 ± 0.002	(2^9^, 2^−17^)	(0.3, 0.7)
5 × 5	2000	98.25 ± 0.85	97.45 ± 0.83	97.85 ± 0.76	0.97 ± 0.008	(2^9^, 2^−17^)	
	261 (A. F. S.)	**98.82** **±** **0.44**	**99.03** **±** **0.81**	**98.93** **±** **0.56**	**0.99** **±** **0.003**	(2^9^, 2^−17^)	(0.1, 0.5)

### Discussion

The results reported in Table 2 indicate that the proposed method for false-positive reduction problem achieved the best recognition rate in terms of Az value, accuracy, and specificity. This result was obtained using MSWLD_24,3_ (4, 4, 5, 5 × 5), SVM with RBF, and feature subset selection. Different parameters involved in the computation of MSWLD, SVM, and Sun’s method for feature selection have significant impact on the recognition accuracy. We performed experiments with different choices of these parameters and found the best set of these parameters. The best parameter values for SVM and Sun’s FSS algorithm are reported in Table 2.

### Comparison

Finally, we give a quantitative comparison with state-of-the-art best method proposed by Lladó et al. [21] in addition to basic WLD. There are two reasons for comparison with this method. First, this method outperforms the most representative state-of-the-art methods (see the comparison given in [21]). Second, LBP histogram used in this method is a texture descriptor like WLD. Table 3 shows the comparison of three methods for false-positive reduction based on MSWLD, LBP, and WLD. Each method was implemented using the same hardware/software environment and was evaluated using the same database. Also note that LBP method was implemented precisely using LBP MATLAB code provided by Ojala et al. [28] and the specifications given in [21], i.e., LBP feature descriptor, were computed by applying ${\text{LBP}}_{8,1}^{u2}$ operator on each of 5 × 5 blocks and ${\text{LBP}}_{{8,{\text{Rsize}}}}^{u2}$ operator on each of central 3 × 3 blocks; according to Lladó et al. [21], this configuration gives the best performance. We used MSWLD_24,3_ (4, 4, 5, 5 × 5) operator for MSWLD feature descriptor and WLD (12, 4, 20) operator for basic WLD feature descriptor; WLD (12, 4, 20) gives the best performance among different combinations of (*T, M, S*). This table indicates that MSWLD-based method outperforms in the reduction in false positives. Note that the difference between the performance of LBP-based method (0.94 ± 0.02) reported in the original work by Lladó et al., and the one (0.92 ± 0.016) shown in Table 1 may be attributed to the selection of ROIs and the evaluation technique; we have used 256 ROIs of true masses and 256 ROIs of suspicious normal tissues; Lladó et al. also used the same number but surely the ROIs are different; it is hardly possible for two different persons to choose the same 256 + 256 cases from a database consisting of more than 2000 cases. The comparison of our method with this method reveals that the proposed method is a better choice for false-positive reduction for a CAD system.

**Table 3:** Comparison between MSWLD, LBP, and basic WLD

	Sensitivity	Specificity	Accuracy	Az
MSWLD	**98.82** **±** **0.44**	**99.03** **±** **0.81**	**98.93** **±** **0.56**	**0.99** **±** **0.003**
LBP	90.35 ± 3.18	93.66 ± 1.18	92.00 ± 0.99	0.92 ± 0.016
Basic WLD	75.62 ± 6.78	69.22 ± 8.60	72.29 ± 2.39	0.72 ± 0.024

Bold values indicate the best results

Now, the question is why MSWLD performs better. The answer to this question is that it has better potential for discrimination of texture microstructures occurring at different locations and with different orientations and scales because it considers the locality, scale, and the orientation of the texture microstructures. Though LBP descriptor encodes the locality and scale of the micropatterns, it does not take into account the orientation of micropatterns.

## Conclusion

We addressed the problem of reducing the number of false positives resulted from the segmentation of mammograms in a CAD system for mass detection. As a solution to this problem, a new method based on MSWLD is proposed; this method recognizes with high accuracy mass and suspicious normal ROIs; in this way, it significantly reduces the number of false positives. MSWLD involves a number of parameters, which has significant impact on the recognition accuracy; a suitable set of these parameters is necessary for optimal recognition rate. We performed experiments to analyze the effect of the parameters and to find the best set of parameters. The best performance is obtained using MSWLD_24,3_ (4, 4, 5, 5 × 5) operator and feature selection. For classification, SVM with RBF was employed, which gave very good detection accuracy. The main credit of the success of the proposed system goes to MSWLD because it encodes the locality, scale, and orientation of texture micropatterns. The direct comparison with a similar state-of-the-art best method based on LBP [21] and indirect comparison with the methods compared with LBP method in [21] show that the proposed method outperforms for false-positive reduction problem. More powerful classifiers like SEL weighted SVM [29] can further improve the detection rate.
